# Lung Function in Traditional Shellfish Divers in Southern Chile—A Cross-Sectional Study

**DOI:** 10.3390/ijerph182010641

**Published:** 2021-10-11

**Authors:** Lorenz Mark, Marie Astrid Garrido, Dennis Nowak, Katja Radon, Laura Wengenroth

**Affiliations:** 1 Institute and Clinic for Occupational, Social and Environmental Medicine, University Hospital, LMU (Ludwig-Maximilians-Universität), 803386 Munich, Germany; lorenzmark99@gmail.com (L.M.); Dennis.Nowak@med.uni-muenchen.de (D.N.); Katja.Radon@med.uni-muenchen.de (K.R.); 2 Comprehensive Pneumology Center (CPC) Munich, German Center for Lung Research (DZL), 80337 Munich, Germany; 3 Center for International Health at Occupational, Social and Environmental Medicine, University Hospital Munich, LMU (Ludwig-Maximilians-Universität), 803386 Munich, Germany; marieastridg@gmail.com

**Keywords:** diver’s lung function, long-term effects, diving, occupational health

## Abstract

The long-term effects of diving on human lung function are controversially discussed. We investigated the lung function of traditional shellfish divers in southern Chile and identified risk factors for reduced lung volumes in divers. In a cross-sectional study, we assessed lung function in traditional shellfish divers and fishermen from two fishing communities. Male divers and fishermen aged 18–60 years were recruited. Participants’ health and diving habits were assessed via standardized questionnaires. Descriptive statistics, chi-squared tests and multiple linear regression models were applied. Through door-to-door sampling, we recruited 112 divers and 63 fishermen (response 67%). Valid spirometries were obtained from 98 divers and 52 fishermen. Divers had higher values of forced vital capacity (FVC, Beta = 0.28, 95% confidence interval (CI): 0.09; 0.47) and forced expiratory volume in 1 s (FEV1, Beta = 0.23, 95%-CI: 0.07; 0.39) compared to fishermen. Among divers, lower values of FVC (Beta = −0.35, 95%-CI: −0.65; −0.05) were found in those with a high diving frequency, while diving depth was associated with higher values of FVC (Beta = 0.28, 95%-CI = 0.04; 0.52). Professional divers had better lung function compared to fishermen. However, among divers, lung function decreased with cumulative diving exposure, warranting approval in future studies to ensure the safety and health of divers.

## 1. Introduction

Divers are exposed to a hyperbaric environment, changing temperature and strenuous exercise. In order to prevent acute and chronic effects of diving on health, such as decompression illness, barotrauma or loss of executive function, divers need to maintain a good health status [1]. In particular, the diversߣ lung function plays an essential role. Several studies have assessed if and how diving activities affect diversߣ lung function in short- and long-term periods, but the results are inconsistent [2,3,4,5,6,7,8,9,10,11,12,13,14,15,16,17,18].

On the one hand, pathological alterations of expiratory flow rates in professional [2,3,4] as well as recreational divers [5] were reported. Chronic exposure to hyperoxia, oxidative and decompression stress might contribute through inflammatory reactions to pathologic alterations in lung tissue and airways, as well as vascular impairments. On the other hand, studies observed increased forced vital capacity (FVC) in divers [6,7,8,9]. Whether increased FVC results from a “training” effect or individuals with an increased FVC are more likely to become divers is still controversially discussed [6,10].

Other investigations in military divers did not detect any differences in divers’ lung function over time [11,12]. However, it must be kept in mind that military divers undergo frequent medical investigations and divers with health problems would presumably have been excluded from diving. Therefore, a healthy worker effect might have been present.

The majority of studies which investigated diversߣ lung function has been performed in recreational divers, professional industrial or navy divers. However, the working conditions of professional artisanal divers differ fundamentally [13,14,15,16]. Apart from older diving equipment, they often dive without proper knowledge of diving physiology, risks and potential long-term health hazards. Many divers work informally in the extraction of seafood. Thus, their daily income depends directly on the quantity of extracted products and their diving performance changes. Studies have observed substantial numbers of dives exceeding the decompression limits: 24% of dives in lobster artisanal fishermen in Mexico and 72% in indigenous fishermen divers in Thailand [13,14].

Approximately 11,000 traditional shellfish divers and 50,000 traditional fishermen work in Chile [19]. Traditional shellfish divers mainly use surface-supplied diving equipment, the so-called “hookah technique”. On board of the fishing boat is a compressor which presses ambient air through a hose and a regulator to the diverߣs mouth. This economic method enables a prolonged diving time. However, the hoses are very sensitive to currents as well as reef edges and are at risk of bending and tearing, and might cause an interruption of the diverߣs air supply. [20] Additionally, global warming, environmental pollution as well as overfishing contribute to the precarious working conditions of traditional shellfish divers [17,18].

The effects of traditional, artisanal shellfish diving on diversߣ lung function has not yet been properly studied. Therefore, the aim of this study was to examine the lung function of traditional Chilean shellfish divers, compare them to a group of local fishermen and identify potential risk factors for impaired lung function.

## 2. Materials and Methods

### 2.1. Study Population and Design

This cross-sectional study examined the lung function of traditional shellfish divers and local fishermen in two different diving communities (Carelmapu, northern shore of Maullín River) in southern Chile. The study took place from September 2017 to October 2018. Male divers and ex-divers aged 18–60 years were included. Fishermen of the same age range who worked as part of the fisher boatߣs crew on deck or as a captain and who had never dived served as a comparison group.

### 2.2. Questionnaire

All interviews and tests were performed by two of the authors (“blinded for review”). We applied questions about socio-demographics and diving exposure (depth and number of dives/year) as well as a screening questionnaire for respiratory symptoms for asthma and chronic bronchitis. We adopted the questions for respiratory symptoms from the European Community Respiratory Health Survey (ECRHS) questionnaire [21].

### 2.3. Spirometry

All lung function tests were performed with the “EasyOne™-Spirometer“ (model 2001, ndd Medizintechnik AG, Technoparkstr. 1, CH-8005 Zürich) by the same two authors (“blinded for review”). According to the guidelines of the American Thoracic Society (ATS) and the European Respiratory Society (ERS), a forced spirometric maneuver was conducted, and the following lung function parameters were measured: forced vital capacity (FVC), forced expiratory volume in 1 s (FEV_1_) and FEV1/FVC ratio. They were either performed in a room at the local harbor or, if the conditions were adequate (privacy without distractions, appropriate space), at the participant’s house.

### 2.4. Ethics

Ethics approval was received. Each participant was informed about the study goals, data protection issues, clinical tests and measurements. An informed consent form had to be signed prior to participation. Those participants who could not read or write were assisted by a relative; in this case, the relative’s signature and the participant’s fingerprint were recorded on the forms. Study participation was voluntarily and in an anonymous form. At any time, the participants were able to resign from the study without any justification.

### 2.5. Statistical Analysis

The statistical software „Stata“ (version 16.1, StataCorp, 4905 Lakeway Drive, College Station, TX 77845, USA) was used for statistical analysis. Descriptive analysis was performed by analyzing arithmetic mean, relative and absolute frequency, as well as standard deviation and 95% confidence interval. Additionally, chi-squared tests and *t*-tests were applied to test differences between divers and fishermen. The level of significance was chosen as *p* < 0.05. Multiple linear regression models were performed to measure the effect of diving on pulmonary function parameters. The results were then adjusted for age, height and smoking status as well as diving exposure (depth and number of dives/year). Spirometry values were used as dependent variables, whereas study group, age, smoking status and height were used as independent variables. Furthermore, we performed a sensitivity analysis in which lung function tests with a lower quality, according to ERS and ATS guidelines, were excluded (Supplementary Tables S1 and S2).

## 3. Results

### 3.1. Descriptive Results

**Figure 1:**
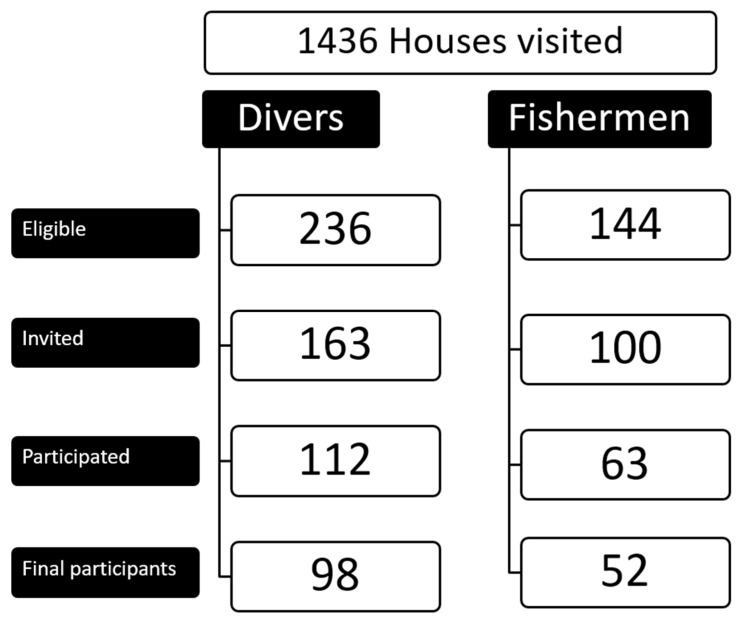
Flow chart of the recruitment process.

In total, we visited 1436 households. A total of 236 divers and 144 fishermen fulfilled the inclusion criteria. We were able to personally invite 163 divers and 100 fishermen, of whom 112 divers (69% response) and 63 fishermen (63% response) participated. Of 166 participants who agreed to perform a spirometry, 150 spirometries met the quality requirements and were used for further analysis (98 from divers and 52 from fishermen, Figure 1). From the 98 divers, 12 were ex-divers (8%) who had stopped diving because of diving accidents (7), other diseases (2), economic reasons (1) or unknown reasons (2). From the 52 fishermen, 7 were ex-fishermen (5%) due to economic (3), health (1) and unknown (3) reasons.

The obtained data presented no statistically significant difference between the group of divers and fishermen considering residence, age, BMI, education and smoking, but divers were a bit smaller (mean = 168 cm) than fishermen (mean = 170 cm, *p* = 0.023; Table 1). Lung function parameters did not differ between divers and fishermen (Table 2). Half of the diving population already had more than 25 years of diving experience (Table 1). Almost half (48%) did 100–200 dives per year. Diving depths during the last 12 months reached down to 30 m for 60% of divers, while 14% dove deeper than 50 m.

### 3.2. Results from Linear Regression Models

Divers had statistically significant higher values for FVC (Beta = 0.28; 95%-CI: 0.09; 0.47) and FEV_1_ (Beta = 0.23; 95%-CI: 0.07; 0.39) than fishermen (Table 3). As expected, lung function significantly decreased with age and slightly, but significantly, increased with height. Smoking was not significantly associated with lung function, while higher education showed a tendency for better lung function, statistically significant for FEV_1_ (Beta = 0.16; 95%-CI: 0.0006; 0.32).

In three separate multiple linear regressions, each including divers only, we observed that divers with a higher diving frequency (100–200 dives in the last 12 months) presented statistically significantly lower values of FVC (Beta = −0.35, 95%-CI: −0.65; −0.05) than divers who reported less than 100 dives in the last 12 months (Table 4). Divers who dove 30 m or deeper showed significantly better FVC (Beta = 0.28; 95%-CI: 0.04; 0.52) than divers who dove less than 30 m. The total number of diving years was not associated with lung function parameters.

In a sensitivity analysis (Supplementary Tables S1 and S2), we performed spirometry according to the ATS and ERS guidelines, allowing even small alterations of pulmonary function to be detected. While significance of results decreased due to decreased sample sizes, the effect sizes remained stable.

## 4. Discussion

To our knowledge, the present study is the first study which examines the lung function of traditional Chilean shellfish divers. In this cross-sectional study, we detected increased lung volumes (FEV_1_, FVC) in divers compared to fishermen. Expiratory flow rates decreased with age but did not differ between divers and fishermen. Among divers, we observed reduced lung volumes (FVC) in divers with a higher diving frequency in the last year and increased lung volumes (FVC) among those who dove 30 m or deeper, compared to those who dove less than 30 m.

The traditional Chilean shellfish divers presented higher lung volumes (FVC and FEV_1_) compared to fishermen. Similar results have been observed in other studies on divers’ lung function [5,6,7]. Whether said results are due to a “training effect” or a process of natural selection remains part of scientific discourse.

On the one hand, the hyperbaric environment, high gas density and increased airway resistance might lead to an increased training stimulus of the respiratory muscles and therefore to higher lung volumes, as described by Davey et al. [8]. Davey et al. analyzed the data of 858 divers from a central diving register in England and found a positive association between diving depth and FVC values, as we did in our study. Furthermore, Fitzpatrick et al. [22] observed the lung function of 43 working divers in a longitudinal study design over 3 years and detected a positive correlation between diving exposure (depth, diving time) and higher lung volumes (FVC, FEV_1_).

On the other hand, studies have been conducted which do not support the hypothesis of a training effect. Voortman et al. [12] studied the pulmonary function of professional divers from the Dutch marine in a retrospective cohort study over 30 years and did not detect any difference between the alteration of divers’ lung function over time compared to non-divers. Therefore, a process of natural selection (people with higher lung volumes tend towards a diving career) might be an alternative explanation.

In the present study, we observed increased lung capacities (FVC) for divers diving 30 m and deeper compared to divers who dove less than 30 m deep, but contrarily, we detected significantly lower FVC values in divers with a higher diving frequency in the last year. Inconsistently, divers with more than 25 years of diving experience did not present statistically lower lung volumes, but the regression coefficient showed a tendency to lower lung volumes. The lack of statistical significance might be due to a small population size. This suggests that frequent exposure to a hyperbaric environment in certain diving populations might lead to impaired lung function values. As previously described, traditional shellfish divers in lower- and middle-income countries tend to lack proper education on diving physiology and additionally tend to work under precarious working conditions compared to standards in high-income countries [13,14,16,17,18]. These factors affect their diving behavior and might lead to a higher risk of pathological long-term effects on their lung function.

We used a cross-sectional study design to evaluate divers’ lung function. Therefore, no causal conclusions can be drawn.

To diminish selection bias, the random walk method was used to recruit participants. Applying this method, we were able to collect a representative sample of two small-scale fishing communities in southern Chile. The fishermen represent a valid comparison group sharing the same environmental exposure factors as divers.

We tried to diminish the healthy worker effect by including former divers and fishermen, although we were only able to include a small sample of ex-workers. For future studies, it would be an interesting approach to compare the divers’ lung function with the one from ex-divers in a larger sample.

In a sensitivity analysis (Supplementary Tables S1 and S2), we performed spirometry according to the ATS and ERS guidelines, allowing even small alterations of pulmonary function to be detected. In order to overcome known short-term effects of depth and cold on pulmonary function after dives [23], we performed all spirometries at least two hours after the last dive.

Some limitations to our study should be considered. We compared divers and fishermen but did not compare them to an existing external reference group. To our knowledge, no suitable external reference group in terms of age and region exists. Furthermore, spirometry possesses limitations, as it is only able to measure certain lung volumes. We did not investigate alterations of the other parts of the pulmonary function, e.g., diffusion capacity of the lungs for carbon monoxide (DLCO), which should be investigated in further studies.

## 5. Conclusions

The majority of recreational and professional divers might be operating within safe diving limits and not accumulate pathologic effects over time. Our study even suggests that divers might have increased lung capacities and higher lung volumes compared to the comparison group. However, we also found that higher diving frequency was associated with reduced lung capacities in divers. This tendency was also shown in divers who had more than 25 years of diving experience, although the results were not statistically significant. These findings might suggest that cumulative exposure to hyperbaric environments in certain diver populations might lead to impaired lung function values. Our findings might provide an important basis for further investigations. To better understand lung function in divers, additional impairments of alveolar diffusion capacity could play a role. New methods have recently been developed to detect the pathological effects of oxygen on the lung function, namely pulmonary oxygen toxicity (POT). In early studies, organic volatile compounds (VOCs) have been used to detect early effects of oxygen toxicity [24]. Although further investigations must prove their validity, they might play an important role in the near future to investigate the full spectrum of aspects of divers’ lung function.

## Figures and Tables

**Table 1:** Demography and health status of the divers and fishermen.

	Divers (N = 98)	Fishermen (N = 52)	*p*-Value ^1^
**Place of Residence**			0.990 ^1^
Carelmapu	83 (85%)	44 (85%)	
Northern shore of Maullín River	15 (15%)	8 (15%)	
**Age (years)**			0.594 ^1^
≤20	1 (1%)	1 (2%)	
20–29	9 (9%)	8 (15%)	
30–39	16 (16%)	11 (21%)	
40–49	34 (35%)	17 (33%)	
50–59	38 (39%)	15 (29%)	
**Height (mean in cm)**	168 (SD ^2^: 5.21)	170 (SD ^2^: 6.73)	0.023 ^3^
**BMI (kg/m²)**			0.805 ^1^
<25	19 (19%)	10 (19%)	
25 ≤ 30	44 (45%)	26 (50%)	
≥30	35 (36%)	16 (31%)	
**Education**			0.770 ^1^
Primary	56 (57%)	31 (60%)	
Secondary	42 (43%)	21 (40%)	
**Smokers**	35 (36%)	20 (38%)	0.740 ^1^
**Diving Years**		n.a.	n.a.
≤25 years	49 (50%)		
>25 years	49 (50%)		
**Diving Frequency ^4^**		n.a.	n.a.
≤100 dives/year	23 (24%)		
100–200 dives/year	46 (48%)		
>100 dives/year	26 (27%)		
**Diving Depth ^5^**		n.a.	n.a.
<30 m	59 (60%)		
30–50 m	25 (26%)		
>50 m	14 (14%)		

^1^*p*-value retrieved from chi-squared test, ^2^ SD: standard deviation, ^3^ *p*-value retrieved from *t*-test, ^4^ data of three participants was missing, ^5^ during the last 12 months.

**Table 2:** Demography and health status of the divers and fishermen.

Lung Function Parameters	Divers ^1^	Fishermen ^1^	*p*-Value ^2^
FVC	4.62 ± 0.71(n = 98)	4.53 ± 0.71 (n = 52)	0.454
FEV_1_	3.62 ± 0.59 (n = 98)	3.54 ± 0.61 (n = 52)	0.401
FEV_1_/FVC	0.78 ± 0.06 (n = 98)	0.78 ± 0.07 (n = 52)	0.693

^1^ means ± standard deviation, ^2^ *p*-value retrieved from *t*-test. Legend: n = number of participants, FVC = forced vital capacity, FEV_1_ = forced expiratory volume in 1 s, FEV1/FVC = Tiffeneau Index.

**Table 3:** Association of diving with lung function retrieved from multiple linear regressions.

	FVC (n = 150)			FEV_1_ (n = 150)			FEV1/FVC (n = 150)		
	Reg.	95%-CI	*p*-Value	Reg.	95%-CI	*p*-Value	Reg.	95%-CI	*p*-Value
**Divers**	0.28	0.09; 0.47	0.005	0.23	0.07; 0.39	0.006	0.003	−0.02; 0.02	0.803
**Age ^1^**									
40–49	−0.43	−0.66; −0.20	<0.001	−0.45	−0.64; −0.25	<0.001	−0.02	−0.05; 0.002	0.067
≥50	−0.60	−0.84; −0.36	<0.001	−0.62	−0.83; −0.42	<0.001	−0.03	−0.06; −0.01	0.007
**Height ^2^**	0.05	0.04; 0.07	<0.001	0.03	0.02; 0.04	<0.001	−0.002	−0.003; −0.0002	0.030
**Smoking**	0.15	−0.04; 0.34	0.116	0.13	−0.03; 0.29	0.101	0.003	−0.02; 0.02	0.729
**Education ^3^**	0.14	−0.05; 0.33	0.145	0.16	0.0006; 0.32	0.049	0.01	−0.01; 0.03	0.242

^1^ Reference category: age ≤ 39 years, ^2^ measured in cm, ^3^ reference category: primary education. Legend: n = number of participants, FVC = forced vital capacity, FEV1 = forced expiratory volume in 1 s, FEV1/FVC = Tiffeneau Index, Reg.: β-regression coefficient, 95%-CI: 95%-confidence interval.

**Table 4:** Association of diving frequency, diving depth and diving years on lung function in divers only ^1^.

	FVC (n = 95)			FEV_1_ (n = 95)			FEV1/FVC (n = 98)		
	Reg.	95%-CI	*p*-Value	Reg.	95%-CI	*p*-Value	Reg.	95%-CI	*p*-Value
**Diving Frequency ^2^**									
101–200	−0.35	−0.65; −0.05	0.024	−0.23	−0.48; 0.02	0.075	0.01	−0.02; 0.04	0.418
>200	−0.25	−0.59; 0.09	0.143	−0.12	−0.40; 0.16	0.405	0.02	−0.01; 0.05	0.255
**Diving Depth ^3^**									
≥30 m	0.28	0.04; 0.52	0.023	0.20	−0.003; 0.40	0.053	−0.004	−0.03; 0.02	0.712
**Diving Years ^4^**									
>25 years	−0.09	−0.41; 0.23	0.576	−0.10	−0.36; 0.16	0.433	−0.01	−0.04; 0.02	0.484

^1^ adjusted for age, height, education and height; retrieved from three separate multiple linear regressions, ^2^ dives/year; reference category: ≤100 dives/year, ^3^ in m; reference category: <30 m, ^4^ reference category: ≤25 years. Legend: m = meter, n = number of participants, FVC = forced vital capacity, FEV1 = forced expiratory volume in 1 s, FEV1/FVC = Tiffeneau Index, Reg. = β-regression coefficient, 95%-CI= 95%-confidence interval.

## Data Availability

Data will be available upon request.

## Supplementary Materials

The following are available online at www.mdpi.com/article/10.3390/ijerph182010641/s1, Table S1: association of diving with lung function retrieved from multiple linear regressions, excluding lung function tests with lower quality according to ERS and ATS guidelines, Table S2: association of diving frequency, diving depth and diving years on lung function in divers only, excluding lung function tests with lower quality according to ERS and ATS guidelines.
