# Supplementary material for: Lung Function in Traditional Shellfish Divers in Southern Chile—A Cross-Sectional Study
Source: Int J Environ Res Public Health. 2021 Oct 11;18(20):10641. doi: 10.3390/ijerph182010641 (PMC8535558; doi:10.3390/ijerph182010641)
Supplement: Supplementary file 1 [file ijerph-18-10641-s001.zip › ijerph-1385636-supplementary.pdf]

International Journal of
Environmental Research and Public Health

MDPI

Supplemental Table S1: Association of diving with lung function retrieved from multiple linear regressions, excluding lung function tests with lower quality according to ERS and ATS guidelines.

	FVC (n=91)			FEV1 (n=91)			FEV1/FVC (n=91)		
	Reg.	95%-CI	p-value	Reg.	95%-CI	p-value	Reg.	95%-CI	p-value
**Divers**	0.21	-0.06; 0.47	0.132	0.10	-0.11; 0.32	0.341	-0.01	-0.03; 0.01	0.344
**Age[1]**									
40-49	**-0.33**	**-0.62; -0.04**	**0.025**	**-0.28**	**-0.51; -0.05**	0.020	-0.002	-0.03; 0.02	0.845
≥50	**-0.57**	**-0.90; -0.25**	**0.001**	**-0.56**	**-0.82; -0.30**	<0.001	-0.03	-0.05; 0.002	0.069
**Height[2]**	**0.06**	**0.04; 0.08**	**<0.001**	**0.04**	**0.03; 0.06**	<0.001	-0.001	-0.003; 0.001	0.249
**Smoking**	**0.25**	**0.002; 0.50**	**0.047**	**0.23**	**0.03; 0.42**	0.025	0.01	-0.01; 0.03	0.481
**Education[3]**	0.19	-0.07; 0.44	0.147	**0.21**	**0.006; 0.41**	0.044	0.02	-0.01; 0.04	0.171

[1]Reference category: age ≤39 years, [2]measured in cm, [3]Reference category: primary education. Legend: n= number of participants, FVC = forced vital capacity, FEV1 = forced expiratory volume in 1 second, FEV1/FVC = Tiffeneau Index, Reg.: β-Regression coefficient, 95%-CI: 95%-Confidence interval.

Supplemental Table S2: Association of diving frequency, diving depth and diving years on lung function in divers only[1], excluding lung function tests with lower quality according to ERS and ATS guidelines

	FVC (n=63)			FEV$_1$ (n=63)			FEV1/FVC (n=63)		
	Reg.	95%-CI	p-value	Reg.	95%-CI	p-value	Reg.	95%-CI	p-value
**Diving frequency[2]**									
101-200	-0.14	-0.54; 0.26	0.490	-0.10	-0.42; 0.21	0.508	0.004	-0.03; 0.04	0.779
>200	0.04	-0.42; 0.50	0.865	0.09	-0.27; 0.45	0.635	0.02	-0.02; 0.05	0.375
**Diving depth[3]**									
≥30m	0.33	0.02; 0.65	0.037	0.26	0.02; 0.51	0.035	0.002	-0.02; 0.03	0.861
**Diving years[4]**									
>25 years	-0.17	-0.58; 0.24	0.410	-0.09	-0.04; 0.23	0.565	0.01	-0.02; 0.04	0.589

[1]adjusted for age, height, education and height; retrieved from three separate multiple linear regressions, [2]dives/year; reference category: ≤100 dives/year, [3]in m; reference category: <30m, [4]reference category: ≤25 years.

Legend: m=meter, n= number of participants, FVC = forced vital capacity, FEV1 = forced expiratory volume in 1 second, FEV1/FVC = Tiffeneau Index, Reg= β-Regression coefficient, 95%-CI= 95%-Confidence interval.
